# Chronic Neutrophilic Leukemia: A Literature Review of the Rare Myeloproliferative Pathology

**DOI:** 10.7759/cureus.15433

**Published:** 2021-06-03

**Authors:** Vishwanath Anil, Harpreet Gosal, Harsimran Kaur, Hyginus Chakwop Ngassa, Khaled A Elmenawi, Lubna Mohammed

**Affiliations:** 1 Internal Medicine, California Institute of Behavioral Neurosciences & Psychology, Fairfield, USA; 2 Internal Medicine, Emergency Medicine, California Institute of Behavioral Neurosciences & Psychology, Fairfield, USA; 3 Medicine, California Institute of Behavioral Neurosciences & Psychology, Fairfield, USA; 4 Surgery, California Institute of Behavioral Neurosciences & Psychology, Fairfield, USA

**Keywords:** chronic neutrophilic leukemia, myeloproliferative disorder, csf3r, jak-stat, setbp1, asxl1

## Abstract

Hematological malignancies often develop due to a vast spectrum of environmental and genetic etiologies. Chronic neutrophilic leukemia (CNL) can be described as a *BCR-ABL1* (Philadelphia chromosome)-negative myeloproliferative neoplastic disease with various genetic mutations that may directly or indirectly play a role in its pathogenesis. A well-established mutation in CNL is the *CSF3R* (a cytokine receptor) which has been incorporated into the diagnostic criteria for the disease. However, evidence of other mutations such as *SETBP1*, *ASXL1*, and *TET2* has also shed more light on the pathogenesis of this condition. Due to the unknown incidence and heterogeneous presentation of the disease, the diagnosis and management are often difficult and lack satisfactory data. The purpose of this review is to yield further insight into a disease that lacks awareness in the medical community. Using PubMed as a database, relevant studies and case reports were reviewed. The data compiled were used to acknowledge the disease in terms of etiology, clinical manifestation, molecular pathogenesis, and available treatment modalities. Though existing treatment modalities have been shown to induce clinical improvement, the outcomes are not reliable, and further research is required to reach a comprehensive “standard of care” for the disease.

## Introduction and background

Chronic neutrophilic leukemia (CNL) is a *BCR-ABL1* (Philadelphia chromosome)-negative myeloproliferative neoplasm, which characteristically presents as a triad of predominantly mature neutrophilic leukocytosis, hepatosplenomegaly, and bone marrow granulocytic hyperplasia [1]. The earliest potential record of the disease was in 1920 when Tuohy reported a case of splenomegaly with polymorphonuclear neutrophil leukocytosis in a 58-year-old woman [2]. Although other case reports had come to light in the following decades, it was finally in 1964 that Tanzer et al. coined the term “chronic neutrophilic leukemia” in The Lancet [3]. Though it was previously considered a diagnosis of the elderly with a median age of onset of 66.5 years, there have been case series showing a younger median age of disease onset of 39 years [4]. Recent literature has also revealed a preponderance for males to develop the condition [5]. The clinical manifestation of CNL shows a vast spectrum of symptoms, including fatigue, weight loss, night sweats, pruritis, gout, easy bruising, and hemorrhagic tendencies [4,5]. Patients may also be completely asymptomatic at diagnosis, and the only manifestation may be an incidental finding of neutrophilic leukocytosis [4]. There have also been reports of grave CNL cases which have presented as cerebral hemorrhage [4,5].

It was finally in 2013 that a landmark study by Maxson et al. discovered the *colony-stimulating factor 3 receptor* (*CSF3R*) oncogene as a key mutation in patients with CNL [6]. It had remained a rare diagnosis of exclusion until the 2016 World Health Organization (WHO) classification recognized the mutations of *CSF3R* as diagnostic for the condition [7]. Various detailed case reports in the past decade have also shown how CNL is linked to other mutations such as *SETBP1*, *ASXL1*, *TET2*, and *calreticulin* (*CALR*) [4,5]. The WHO diagnostic criteria of 2016 for CNL was formulated to rule out the likelihood of secondary or clonal neutrophilia, which may be seen in myeloid malignancies apart from CNL [8]. The management of CNL presently does not have an established standard of care [9]. Drugs such as hydroxyurea are used as first-line chemotherapy but have not always provided a stable response [10]. Though it has been shown to bring about remission, the indication for allogeneic stem cell transplant (ASCT) lacks sufficient data [1,4,5]. Targeted therapies such as dasatinib and ruxolitinib (JAK-1/2 inhibitor) are still being studied [11]. Other first-line treatment modalities include interferon-alpha and splenectomy, but they also lack adequate evidence of therapeutic progress [1,4].

Though the evidence uncovered about genetic mutations associated with CNL has been substantial in the past decade, the exact mechanism involved in leading to CNL remains unknown [12]. These major gaps in knowledge may be due to the complexity of the signaling pathways being studied and the limitations of the technology used to study them [12]. With increasing research into molecular pathogenesis, we have been able to identify several mutational combinations that are relevant to developing therapeutic solutions [9]. Nevertheless, the challenge remains in transforming the existing data into a standard prognostic schema and developing meaningful therapeutic advancement [9]. There also exists a need to study the disease further to understand the exact reason behind its poor prognosis and limited response to treatment modalities [1]. This review summarizes the available data on CNL with special emphasis on the disease’s clinical manifestations and molecular pathophysiology. The recent advances in treatment modalities and subsequent prognostic benefits have also been studied at length.

Search strategy

Using the keywords mentioned in Table 1, a comprehensive search was conducted to identify the studies analyzing the clinical manifestations of CNL using PubMed as the primary database. In addition to the primary aim of the study, the pathophysiology and the management modalities of CNL have also been extensively discussed and reported. The data assessed were acquired from studies published in the last 10 years. There was no restriction of study type, i.e., traditional reviews, systematic reviews, clinical trials, case-control studies, and cohort studies. Only studies for which free full texts were available on PubMed were analyzed. Studies were not refined based on age or ethnicity. The search was not limited to the basis of demography. All the data reviewed were from studies published in the English language. Data published in the last 10 years were reviewed and the results obtained are summarized in Table 1.

**Table 1:** Keywords and search results. MPN: myeloproliferative neoplasm; JAK: Janus kinase; STAT: signal transducer and activator of transcription proteins; *CSF3R*: *colony-stimulating factor 3 receptor*; *ASXL1*: *ASXL transcriptional regulator 1*; CNL: chronic neutrophilic leukemia; *SETBP1*: *SET binding protein 1*

Keyword	Database	No. of results
MPN	PubMed	6,087
JAK-STAT	PubMed	3,778
CSF3R	PubMed	1,100
ASXL1	PubMed	519
CNL	PubMed	206
SETBP1	PubMed	126
MPN and CNL	PubMed	68
CNL and CSF3R	PubMed	58
CNL and SETBP1	PubMed	20
CNL and ASXL1	PubMed	11

## Review

In the following section, we shall look into the epidemiological data, various clinical features, and the appropriate diagnostic approach to CNL. The current diagnostic criteria and existing treatment modalities have also been reviewed.

Epidemiology

The WHO included CNL in their Classification of Neoplastic Diseases in 2001 [13]. Though the disease is rare, there has been a steady rise in the case count of CNL since the 90s [9]. In 1996, there were less than 100 cases reported, but now there are over 200 documented cases as per the National Cancer Institute Surveillance, Epidemiology and End Results Program (SEER) report of 2019; yet, the true incidence of CNL remains unknown [9]. CNL was considered to be a diagnosis of the elderly population, with data showing that 88% of cases were over the age of 50 with a median age of onset of 66.5 years in a large WHO-defined case series [4]. It was Ouyang et al. who provided data showing a younger median age of disease onset of 39 years through their 10 patient cohorts [14]. The most frequently documented mutation in CNL is the proximal membrane point mutation T618I of *CSF3R*, seen in 88% of the cases [11]. The youngest patient to meet the diagnostic criteria for CNL on record is an 11-year-old girl with a *CSF3R* T618I germline mutation who has remained stable with leukocytosis for 11 years [15]. The sex distribution of CNL was thought to be equal until recent literature from a *CSF3R*-mutated cohort revealed a proclivity for males to develop the condition [4,5].

Though the course of the illness may be variable, the most common causes of patient deterioration are refractory neutrophilia, transformation to acute myeloid leukemia (AML), and aggravated organomegaly [5,9]. Intracranial hemorrhage, blast transformation, and chemotherapy/transplant regimen-related toxicity are the most common causes of death in CNL [4,5]. The median time for the transformation to AML is 21 months, and the median survival is 23.5 months [5]. National statistics reveal that CNL has a short median overall survival (OS) of 1.8 years and 2.2 years according to the SEER and the National Cancer Database (NCDB), respectively [1]. Recent reports still show that CNL is an incredibly rare disease, and there has not been a rise in the incidence of CNL following the discovery of *CSF3R *mutation [1].

Disease features and diagnosis

The clinical picture of CNL has been heterogeneous in nature, with presentations ranging from an incidental finding of neutrophilic leukocytosis on routine blood analysis to a symptomatic spectrum including weight loss, night sweats, fatigue, pruritis, easy bruising, hepatosplenomegaly, and gout [4,5]. The majority of patients often remain asymptomatic at the time of diagnosis [4]. The most commonly reported symptoms are fatigue and hepatosplenomegaly, while the development of lymphadenopathy has remained uncommon in the presentation of CNL [4,5]. Exclusion of other causes of neutrophilia is required for the diagnosis of CNL [16]. Reactive neutrophilia may be present in infectious, inflammatory, and neoplastic conditions [8]. Plasma cell neoplasms may also present with secondary neutrophilia, whereas solid malignancies of the lung, connective tissues, and urogenital tract can lead to the development of paraneoplastic hyperleukocytosis [8,16]. Mature neutrophils are also a feature of other myeloid malignancies such as atypical CML (aCML) and chronic myelomonocytic leukemia (CMML) [8]. It is also important to differentiate CNL from other myeloproliferative neoplasms (MPN) such as polycythemia vera (PV), primary myelofibrosis (PMF), and essential thrombocythemia (ET) [5,9]. A commonly observed complication is the presence of hemorrhagic tendencies, with a considerable fraction of patients developing cerebral hemorrhage [4,5]. Evidence revealed by Bohm shows that cerebral hemorrhage was the most common cause of death in the described case series of 14 patients [17]. The pathophysiology of this bleeding diathesis can be traced to the commonly observed development of acquired von Willebrand disease, platelet dysfunction, and thrombocytopenia or may even be due to the neoplastic infiltration of the vessel wall [4].

Chronic, sustained, mature neutrophilia is pathognomonic of CNL [4,5,15]. The noteworthy absence of eosinophilia, basophilia, and monocytosis is a distinguishing feature from the diagnosis of chronic myeloid leukemia (CML) [4]. CNL also predisposes patients to the development of thrombocytopenia and mild anemia [4]. Elevated levels of leukocyte alkaline phosphatase (LAP) is yet another distinguishing factor from CML, which generally presents with low LAP values [9]. Vitamin B12 levels may also appear elevated due to the granulocytic release of transcobalamin [18]. Low levels of granulocyte colony-stimulating factor (G-CSF) have also been identified in cases of CNL, but its practical application in diagnosing CNL is limited [4,5]. Dohle bodies and neutrophil toxic granulations are not uncommon in CNL, though they are nonspecific observations in neutrophilic leukemoid reaction [4,18]. Hypercellularity of bone marrow (cell-to-fat ratio of approximately 90:10) is a diagnostic criterion in CNL due to the expansion of neutrophilic granulopoiesis [19]. Myeloblasts are <5% without the presence of Auer rods [4]. Bone marrow fibrosis may develop in CNL, and it plays an important role in the pathophysiology and prognosis of the disease [20]. Presented below in Table 2 are the diagnostic criteria for CNL as stipulated by the WHO.

**Table 2:** Diagnostic criteria for chronic neutrophilic leukemia. Adapted from Arber et al. [21]. WHO: World Health Organization; PB: peripheral blood; PV: polycythemia vera; ET: essential thrombocythemia; *BCR-ABL1*: Philadelphia chromosome; CML: chronic myeloid leukemia; PMF: primary myelofibrosis; *PDGFRA/B*: *platelet-derived growth factor receptor A/B*; *PCM1*: *pericentriolar material 1*; JAK2: Janus kinase 2; *FGFR1*: *fibroblast growth factor receptor 1*; *CSF3R*: *colony-stimulating factor 3 receptor*

WHO revised diagnostic criteria for chronic neutrophilic leukemia (2016)
1. PB leukocytosis ≥25 × 10^9^/L
Segmented neutrophils + band forms ≥80% of leukocytes
Precursors of neutrophils (metamyelocytes, promyelocytes, and myelocytes) <10% of leukocytes
Myeloblasts rarely identified
Monocyte count <1 × 10^9^/L
Absence of dysgranulopoiesis
2. Hypercellular bone marrow
Neutrophil granulocytes increase in number and percentage
Normal-appearing maturation of neutrophils
Myeloblasts <5% of the nucleated cells
3. Does not meet the WHO criteria for PV, ET, *BCR-ABL1*-positive CML, or PMF
4. No rearrangement of *PDGFRA/B*, or *PCM1-JAK2*, or *FGFR1*
5. *CSF3R* T618I or other activating *CSF3R* mutations are identified
Or
When there is a lack of a *CSF3R* mutation, persistent neutrophilia for at least 3 months, splenomegaly, and absence of identifiable cause of reactive neutrophilia, including an absence of a plasma cell neoplasm or, if identified, demonstration of myeloid cell clonality by molecular or cytogenetic analysis

Molecular pathogenesis

The G-CSF is a hematopoietic cytokine involved in regulating granulopoiesis and granulocyte differentiation through the dimerization of G-CSF receptor (e.g., *CSF3R*) [22]. Mutations of *CSF3R *are found in a spectrum of myeloid disorders and malignancies [12]. The role of G-CSF in granulopoiesis has been demonstrated with murine study models [9,22]. G-CSF receptor belongs to the type 1 superfamily of cytokine receptors and appears to be a single cell-surface receptor [9]. The cytoplasmic domain of the receptor contains different functional regions, while the membrane-proximal region is involved in mitogenic signaling, and the carboxy-terminal regulates proliferation and maturation signaling [9,13]. There are various pathways through which G-CSF exerts its effects, namely, the Janus kinase-signal transducer and activator of transcription (JAK-STAT), nonreceptor tyrosine kinase (SYK), PI3K/Akt pathways, Src family kinases (e.g., LYN), and Ras/Raf/MAP kinases [5,6,9,11,12,15].

Though *CSF3R* mutations have been observed in other myeloid disorders such as hereditary chronic neutrophilia, severe congenital neutropenia (SCN), and in some myeloid leukemia as well, it was in 2013 that a landmark discovery by Maxson et al. identified the disease defining *CSF3R* mutations in CNL, thus marking a turning point in the molecular pathogenesis of the disease [6,9]. With this study, *CSF3R* mutations were identified in 89% of the cases of CNL [9]. The mutational variants of *CSF3R* are the membrane-proximal mutations, T618A and T618I point mutations, and the nonsense or frameshift mutations observed in the *CSF3R* cytoplasmic tail [6,9,22]. *CSF3R* T618I has been observed to undergo vertical transmission, as evidenced by a case report illustrating a familial type of CNL [23]. Two distinct classes of *CSF3R* mutations have also been identified in CNL; one is truncation mutations resulting from the Src family-TNK2 kinases dysregulation and the other is membrane proximal mutations that lead to JAK family kinase dysregulation [5,6,12]. These two types of *CSF3R *mutations cause a difference in response to classes of tyrosine kinase inhibitors [6]. *CSF3R* truncation mutations show sensitivity to dasatinib, while *CSF3R* membrane proximal mutations display an excellent response to ruxolitinib [24].

Fleischman et al. established the ability of *CSF3R* T618I, which is the most common *CSF3R* mutation, to drive leukemogenesis in CNL using a murine model of a bone marrow transplant [25]. It was also identified that the *CSF3R* T618I mutation exerted its effect through the JAK-STAT signaling pathway; hence, the splenomegaly and granulocytosis were responsive to treatment with ruxolitinib, a JAK inhibitor [5,6,9,11]. *CSF3R* T640N is another transmembrane domain mutation that expresses a leukemogenic potential and therapeutic response to ruxolitinib [9]. Though the *CSF3R* T618I mutation has become a diagnostic mutation in CNL, it has been reported that additional mutations are essential for the progression of the disease [15]. This observation can be compared to the secondary genetic mutations observed in the pathogenesis of CML [15]. Pardanani et al. conducted a follow-up study to analyze *CSF3R* in a subset of WHO-defined patients, and their data were able to endorse the *CSF3R* T618I mutation as both a specific and sensitive genetic marker for the diagnosis of CNL [26]. Rohrabaugh et al. further performed studies to establish the ability of *CSF3R* compound mutations in the induction of aggressive leukemia in a murine model [27]. Their data established the potential for enhanced MAP kinase signaling observed in myeloid malignancies harboring *CSF3R* mutations and suggested the development of resistance to JAK inhibitors in these patients [9,27].

CNL has also been linked to other genetic mutations such as the *SET binding protein 1* (*SETBP1*), epigenetic modifiers (e.g., *ASXL1*, *TET2*), and spliceosome proteins (e.g., SRSF2, U2AF1) [4,5,13]. The evidence supporting the link between these mutations and development CNL suggests more complex pathogenesis than previously assumed [13,23]. It also points to the potential of preleukemic clonal cell phenomenon related to age which could later lead to the development of CNL [13]. *SETBP1* mutations were initially identified in patients with aCML and were an indicator for a worse prognosis [4,13]. Subsequently, *SETBP1* mutations were discovered in CNL patients as well, especially in those who harbored the *CSF3R* mutation [4,5,13]. Present evidence suggests that *SETBP1* mutations do not affect the prognosis in CNL [28]. A potential pathogenetic role has been described for *SETBP1* in the evolution of CNL into blast phase disease [29]. SET has now been identified as a negative regulator for the tumor suppressor, protein phosphatase 2A (PP2A), which plays an important role in inhibiting cell proliferation [4,13]. A fraction of patients with AML also possesses an overexpression of *SETBP1*, which points to poor disease outcomes [13]. CMML, MDS, and juvenile myelomonocytic leukemia are other myeloid malignancies that possess *SETBP1 *mutations [13,28].

Mutations in genes involved in the regulation of epigenetic processes in myeloid malignancies have also been identified [13]. The *ASXL transcriptional regulator 1* (*ASXL1*) mutation has been observed in CNL patients, and its presence has been associated with a poor prognosis [5,9,13]. ASXL1 is involved in the regulation of histone modification and plays a potential role in the transformation of CNL into CMML [9,29]. The *tet methylcytosine dioxygenase 2* (*TET2*) is another mutation that is observed in CNL but with a comparatively lesser prevalence [5,9,13]. Mutations in the spliceosome complex have also been observed in *U2AF1* and *SRSF2* genes in a fraction of patients [13]. The *CALR* mutation is yet another mutation that was initially described in 2014 [30]. It was later studied in the Mayo Clinic but is yet to yield any therapeutic or prognostic significance [9]. Other mutations detected occur in *JAK2*, *KRAS*, *CBL*, and *NRAS*, which are genes of signaling pathways [13]. There is also a report of the missense mutation of *runt-related transcription factor 1* (*RUNX1*) being detected in the setting of blast transformation of CNL [31]. *RUNX1 *is a transcription factor that is vital to the development of stem cells [31]. Another report points to abnormalities of the X chromosome, i.e., loss or gain of the X chromosome, associated with the rapid progression of the disease [20]. This finding holds true for other hematological malignancies as well [20].

Management

The WHO-revised diagnostic criteria for CNL have been used to establish the diagnosis of the disease conclusively since 2016 [4,21]. A full blood count is often the basic diagnostic test that shows the peripheral leukocyte count above the diagnostic threshold, followed by a blood film that reveals marked neutrophilia [24]. Abdominal ultrasonography can be used to confirm splenomegaly [4,24]. Bone marrow biopsy is also indicated to identify hypercellular bone marrow with supporting diagnostic features [4,5,24]. *BCR-ABL1* mutation can be ruled out using the reverse transcription-polymerase chain reaction (RT-PCR) and allele-specific PCR can be employed to rule out *JAK2 V617F* [24]. The presence of *CSF3R *mutation or other activating *CSF3R *mutations has become one of the five diagnostic criteria for CNL [4,21]. The mutation of *CSF3R *is often identified using deep-sequencing techniques [24]. The next-generation sequencing (NGS) panels are considered to be superior to Sanger sequencing as the former requires lesser time, resources, and a smaller quantity of deoxyribonucleic acid to produce a comprehensive genomic profile of cases [24].

Though a “standard of care” has not been established in the case of CNL, hematopoietic stem cell transplantation (HSCT) has become a recommendation in eligible patients [1,5,9]. There is currently no data showing improvement in OS using a therapeutic agent [9]. Since the 1970s, splenic irradiation and splenectomy have been indicated in the palliative care of CNL patients who present with symptomatic splenomegaly [9]. However, reports of postoperative progression of neutrophilia have led to a halt in the recommendation of splenectomy as a treatment of choice [1,5,9]. Chemotherapeutic agents are yet another treatment modality that has been indicated in CNL, with hydroxyurea being the agent that has most commonly been used [1,9,10]. A median duration of therapy lasting 12 months has been reported to show a significant response in about 75% of the patients [9]. However, most studies have shown that treatment with hydroxyurea alone is insufficient, with patients requiring second-line and even third-line agents to be added to the treatment regimen [9,10]. Interferon-alpha is the only agent that has been known to offer potential long-term remissions, though the data have been limited to case report evidence [9].

A JAK 1/2 inhibitor, ruxolitinib, has the ability to suppress the oncogenic JAK-STAT pathway signaling and could, therefore, produce therapeutic value in the management of CNL [4-6,11]. A patient detected with the *CSF3R* T618I in the study by Maxson et al. showed a significant clinical response following treatment with ruxolitinib for 11 months [6]. However, there are numerous cases in which ruxolitinib alone has had an initial response but subsequently failed and required additional therapy [9]. Nonetheless, ruxolitinib has been considered to be a rational approach for patients who are ineligible for HSCT [9]. Depending on the class of *CSF3R* mutation, a difference in response has been observed to the tyrosine kinase inhibitors [6]. CNL patients with *CSF3R *truncation mutation elicit sensitivity to dasatinib, whereas *CSF3R *membrane-proximal mutation responds to ruxolitinib [6,13,24]. It is also worth noting that dasatinib therapy has been shown to be ineffective in a case of *CSF3R *compound mutation which consists of a primary membrane-proximal mutation and a secondary truncation mutation while therapy with ruxolitinib has been efficacious [11].

**Figure 1:**
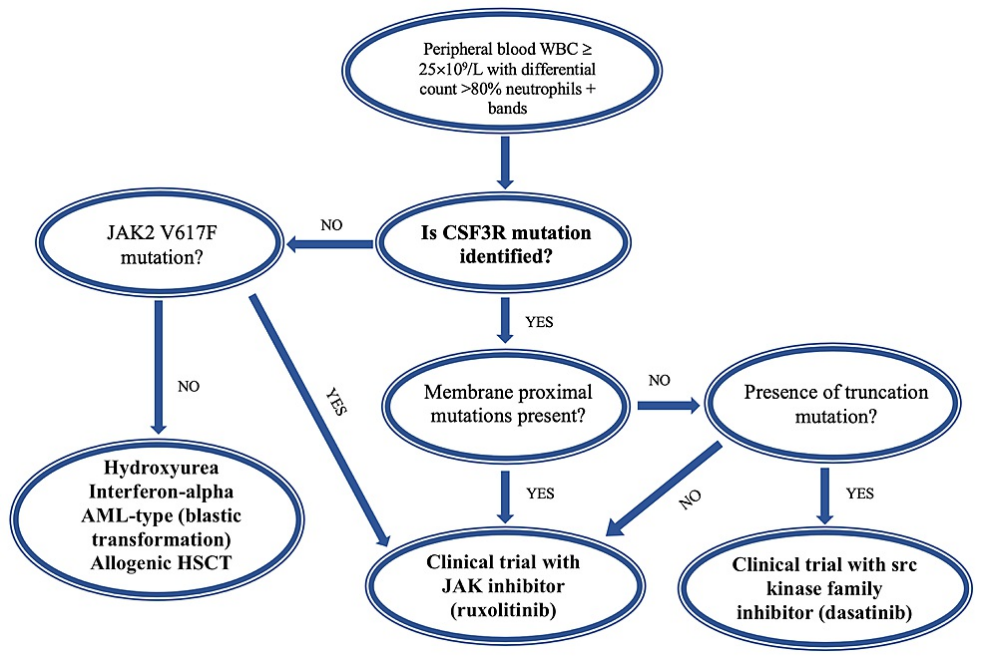
The diagnosis and management algorithm for chronic neutrophilic leukemia. Adapted from Menezes and Cigudosa [5]. Note: In a patient who develops predominantly neutrophilic granulocytosis, the presence of *CSF3R *mutation in the proximal membrane is often sufficient to make the diagnosis of CNL. HSCT: hematopoietic stem cell transplantation; WBC: white blood cells; JAK2: Janus kinase 2; *CSF3R*: *colony-stimulating factor 3 receptor*; AML: acute myeloid leukemia; CNL: chronic neutrophilic leukemia

The standard induction chemotherapy with cytarabine and anthracycline antibiotics such as daunorubicin does not show clinical remission in CNL, though there has been one case reported in which the blast phase reverted to a second chronic stable phase using the “7+3” chemotherapy regimen [9]. Hasle et al. in 1996 were the first to report the utilization of HSCT to attain long-term remission in CNL [32]. Evidence shows that 71% of patients who underwent HSCT during the chronic phase of disease had remissions lasting over seven months, while those who underwent transplantation in the accelerated phase reported a shorter duration of remission [5,9]. A study in Japan revealed a one-year OS rate of 40% in CNL patients who were treated with allo-HSCT [33]. Post-HSCT, the monitoring of *CSF3R *mutation can be applied as a biomarker for the relapsing disease [34]. Mitogen-activated protein kinase kinase (MEK) inhibitors such as trametinib have been speculated to be a potentially beneficial drug due to the discovery of *NRAS *gene mutations in a subset of CNL patients [9]. Figure 1 shows an algorithm for the diagnostic and therapeutic approach to CNL.

Limitations

The scope of this review poses many limitations as the studies included for analysis have been derived within a specific time frame and language, i.e., the last 10 years and English, respectively. Furthermore, the diagnosis of CNL has remained a rare entity in the field of medicine and its definition has dynamically changed in the last two decades. The data presented in this review rely heavily on unique case reports and reviews. Even with the landmark discovery of the *CSF3R *mutation, the complete pathogenesis of the disease remains unclear as more and more genetic factors are being reported, with evidence pointing to potential causation. Another challenge faced while trying to outline the management of this condition is the lack of significant data on patient follow-up and prognosis with different treatment modalities. The evidence on management was extracted from data reported through individual case reports. In preparing this review, it has become abundantly clear that the pathological entity described as CNL requires further research and resources to establish comprehensive knowledge about the illness.

## Conclusions

CNL is a rare myeloproliferative syndrome that can have a vast array of presenting clinical features. This makes the diagnosis of the condition a challenge, especially considering that the disease had remained a diagnosis of exclusion until less than a decade ago. The molecular pathogenesis was also unclear until a landmark study in 2013 revealed direct causation between the mutation of *CSF3R* and the disease. This discovery has led to the incorporation of *CSF3R* mutation into the WHO diagnostic criteria for CNL which was published in 2016. Following this discovery, other genetic mutations have also been identified and classified, such as the *SETBP1*, *ASXL1*, and *TET2*. The pathways through which these mutations cause their effect have also been identified and have become an aid to theorizing and proving the efficacy of various chemotherapeutic and immunologic agents in the management of CNL. Ruxolitinib and dasatinib are major examples of drugs that have been proven to work in various subsets of patients depending upon the genetic mutation identified in them. Other treatment modalities that are currently being used have not shown reliable long-term results and a “standard of care” is yet to be established. It is pertinent that more research and resources be put into understanding this disease on a molecular level as it may reveal the key to discovering a reliable therapeutic technique for this illness. Breakthroughs in molecular genetics are helping the search for more comprehensive answers to unlocking the unknowns of CNL.
